# Targeting Chondroitin Sulfate Reduces Invasiveness of Glioma Cells by Suppressing CD44 and Integrin β1 Expression

**DOI:** 10.3390/cells10123594

**Published:** 2021-12-20

**Authors:** Yin-Hung Chu, Wen-Chieh Liao, Ying-Jui Ho, Chih-Hsien Huang, To-Jung Tseng, Chiung-Hui Liu

**Affiliations:** 1 Department of Anatomy, Faculty of Medicine, Chung Shan Medical University, Taichung 402306, Taiwan; spitwater9@gmail.com (Y.-H.C.); khrnangel@gmail.com (W.-C.L.); fifiness2@gmail.com (C.-H.H.); tjtseng@csmu.edu.tw (T.-J.T.); 2 Department of Medical Education, Chung Shan Medical University Hospital, Taichung 402306, Taiwan; 3 Department of Psychology, Chung Shan Medical University, Taichung 402306, Taiwan; joshuayjho@gmail.com

**Keywords:** glioma, chondroitin sulfate, CHSY1, integrin β1, CD44, therapeutic peptide

## Abstract

Chondroitin sulfate (CS) is a major component of the extracellular matrix found to be abnormally accumulated in several types of cancer tissues. Previous studies have indicated that CS synthases and modification enzymes are frequently elevated in human gliomas and are associated with poor prognosis. However, the underlying mechanisms of CS in cancer progression and approaches for interrupting its functions in cancer cells remain largely unexplored. Here, we have found that CS was significantly enriched surrounding the vasculature in a subset of glioma tissues, which was akin to the perivascular niche for cancer-initiating cells. Silencing or overexpression of the major CS synthase, chondroitin sulfate synthase 1 (CHSY1), significantly regulated the glioma cell invasive phenotypes and modulated integrin expression. Furthermore, we identified CD44 as a crucial chondroitin sulfate proteoglycan (CSPG) that was modified by CHSY1 on glioma cells, and the suppression of CS formation on CD44 by silencing the CHSY1-inhibited interaction between CD44 and integrin β1 on the adhesion complex. Moreover, we tested the CS-specific binding peptide, resulting in the suppression of glioma cell mobility in a fashion similar to that observed upon the silencing of CHSY1. In addition, the peptide demonstrated significant affinity to CD44, promoted CD44 degradation, and suppressed integrin β1 expression in glioma cells. Overall, this study proposes a potential regulatory loop between CS, CD44, and integrin β1 in glioma cells, and highlights the importance of CS in CD44 stability. Furthermore, the targeting of CS by specific binding peptides has potential as a novel therapeutic strategy for glioma.

## 1. Introduction

Chondroitin sulfate (CS) is a sulfated glycosaminoglycan (GAG) that is distributed on the cell surface and in the extracellular space. CS chains are covalently linked to a core protein known as CS proteoglycan (CSPG), thereby endorsing various biological functions to CSPGs by maintaining the tissue physical structure to mediate protein–protein interactions between the cells and the extracellular matrix (ECM) [1,2]. Previous studies have shown that CS is the most abundant GAG in the ECM of the central nervous system (CNS) [3,4]. Typically, the CSPGs in the CNS participate in neural development and support the microenvironment for neural growth and repair [3,4,5]. Furthermore, they are also known to modulate neural stemness phenotypes, cell proliferation, and differentiation in the CNS. Furthermore, various reports have shown excessive CSPG accumulation during CNS injuries and tumors [6,7,8]. CNS injuries have been documented to activate astrocytes and microglia to construct CS-rich glial scars, and the overproduced CSPGs are usually considered suppressors of the neurite outgrowths that impede neural rejuvenation [9]. Many ectopically expressed CSPGs have also been proposed to promote CNS tumor formation and disease progression. For instance, over 60% of patients with glioblastoma (GBM) overexpress CSPG4, thereby serving as an important prognostic factor. Moreover, various studies have investigated the potential of CSPG4 as a target for chimeric antigen receptor T (CAR-T) cell therapy in mouse GBM models [10,11,12,13]. Additionally, glioma-derived versican has been suggested to promote tumor expansion by stimulating Toll-like receptor 2 signaling in tumor-associated macrophages [14]. These findings thus indicate that abnormal CSPGs are crucial in the pathogenesis of CNS-associated diseases and conditions. However, the functions of their CS side chains remain fundamentally unexplored.

The biosynthesis of CS chains on CSPG is initiated by the linkage of N-acetylgalactosamine (GalNAc) to a tetrasaccharide structure by CS GalNAc-transferases (CSGALNT1 and CSGALNT2). The subsequent elongation/polymerization of CS chains is catalyzed by a group of enzymes (CHSY1, CHPF, CHPF2, and CHSY3), which possess both β1–3 glucuronosyltransferase and β1–4 N-acetylgalactosaminyltransferase activities [3,4,15]. Our previous studies have identified that CHSY1 is frequently upregulated in tumor tissues such as human glioma and hepatocellular carcinoma. Moreover, the upregulation of CHSY1 not only enhances CS formation on cancer cells, but is also positively associated with poor patient outcomes [16,17]. However, the specific CSPGs involved in the CHSY1-regulated malignant phenotypes of cancer cells are yet to be explored.

CSPGs are known to interact with several transmembrane proteins, such as the integrins and receptor tyrosine kinases which modulate the cell signaling associated with the glioma cell proliferation, invasion, migration, angiogenesis, and metastasis [18,19,20,21]. Integrins are crucial transmembrane proteins that connect cells and their microenvironment, and are composed of different subtypes of α and β subunits with specific binding abilities to ECM elements [22,23,24]. Interestingly, while integrins on their own are not considered as drivers of tumorigenesis, they have been shown to cooperate with other oncogenes and assist cancer cell invasiveness and angiogenesis in various tumors [22]. In fact, multiple reports have demonstrated that several integrin heterodimers, such as α6β4, α5β1, α2β1, and αvβ6, are upregulated in human gliomas [25,26,27]. Notably, the integrins were found to be closely associated with CS chains in cancer cells, and blocking CS formation resulted in the inhibition of downstream integrin signaling [12,28]. However, which CSPGs are involved in this phenomenon remains unknown.

Herein, we propose for the first time that distinct CS distribution patterns are associated with high-grade gliomas. Moreover, the key CS synthase, CHSY1, regulates the expression of integrins and modifies CS chains on CD44 in glioma cells. Importantly, using a CS-binding peptide that suppresses glioma invasiveness and inhibits integrin and CD44 cooperation, we highlighted the therapeutic potential of targeting CS in human glioma.

## 2. Materials and Methods

### 2.1. Cell Culture and Transfection

The information and source of GBM cell lines GBM8401, GBM8901, DBTRG, Ln18, U118, U251, A172, and GL261 are listed in Table S1. Cell lines were cultured in DMEM with 10 % fetal bovine serum (FBS), 2 mM glutamine, 100 U/mL penicillin, and 100 μg/mL streptomycin (Gibco™, Waltham, MA, USA). All cell cultures were maintained at 37 °C in a humidified atmosphere of 5% CO_2_. CHSY1-pcDNA3.1 plasmid or empty vectors were transfected to cells using TOOLstrong Transfection Reagent (BIOTOOLs, New Taipei City, Taiwan) and selected with 600 μg/mL of G418 (Gibco™, Waltham, MA, USA). With a gene silencing assay, *CHSY1* ON-TARGETplus SMARTpool siRNA or non-targeting control siRNA (Dharmacon, Lafayette, CO, USA) were transfected to cells using Lipofectamine RNAiMAX (Invitrogen, Waltham, MA, USA). The siRNA sequence is listed in Table S2.

### 2.2. 6-O-Sulfated CS (C6S) Binding Peptide

We referred to previous reported C6S binding peptide sequences which can specifically block C6S activity [29,30,31]. This N-terminal biotinylated C6S specific binding peptide (C6S-p; Biotin-EKRIWFPYRRF) and scramble peptides (Biotin-RPWREKIFYRF) were synthesized by Kelowna International Scientific Inc., New Taipei City, Taiwan. The peptides were purified by HPLC (>98% in purity), and confirmed by mass spectrometry (MALDI).

### 2.3. Tissue Array, Immunohistochemistry, Immunofluorescence, and Confocal Microscopy

To analyze CS distribution in human glioma, we purchased paraffin-embedded human glioma tissue microarrays from Shanghai Outdo Biotech. Tissue slides were blocked with 5% bovine serum albumin with 0.1% Triton X-100 (Sigma-Aldrich, Burlington, MA, USA) for 2 h. After blocking, CS56 antibody (1:200) was added for 16 h at 4 °C. Biotinylated goat anti-mouse IgM antibody and avidin–biotin complex kits (Vector Laboratories, Cat. PK-4010, Burlingame, CA, USA) were used. The specific immunostaining was visualized with 3,3-diaminobenzidine and counterstained with hematoxylin (Sigma-Aldrich, Burlington, MA, USA). The signal intensities were graded by microscopy by two scorers blinded to the clinical parameters (0: negative; +1: <20%; +2: 20–50%; +3: >50%).

For immunofluorescence, cells were seeded on the cover slide and treated with C6S-p (100 μg/mL) for 20- or 120-min. Streptavidin-conjugated Alexa Fluor 594 (Invitrogen, REF S11227, Waltham, MA, USA) was used for biotin staining. FITC anti-human CD44 and PE anti-human CD29 antibodies (BioLegend, San Diego, CA, USA) were used. Confocal images were captured using a Leica TCS SP8 confocal microscope. Each confocal image from the cell slides was stacked, and five scan images were merged into one graphic. The total thickness in one capture was 1.5 μm.

### 2.4. Cell Adhesion Assay

96 well plates were coated with collagen I, collagen IV, fibronectin, laminin, or BSA at concentrations of 5 µg/mL in PBS for 16 h, and then blocked with 1% BSA in PBS at 37 °C for 2 h. Glioma cells were detached by 10 mM EDTA, and 2 × 10^4^ cells in 100 µL serum-free DMEM per well were allowed to attach to coated plates at 37 °C for 1 h. The plates were washed once with PBS and stained with crystal violet. The attached cells were measured by spectrophotometer at 562 nm. Independent experiments were repeated for three times.

### 2.5. Cell Migration and Invasion Assay

The cell migration assays were measured by a wound healing assay and transwell inserts (Corning, Glendale, AZ, USA). For the wound healing assay, cells were seeded in 6-well culture plate in over 95% confluence, and scratched using a 1 mL pipette tip to create a gap. A total of 100 μg/mL C6S-p and scramble peptide were added into the medium after the scratch was made. The length of the gap was measured at 0 h, 12 h, 24 h, and 36 h.

Cell transwell assays were performed on transwell inserts (Corning, Glendale, AZ, USA), with or without Matrigel (BD Biosciences, Franklin Lakes, NJ, USA) on the insert filters (pore size 8 μm), and the inserts were set into 24-well plates for the experiments. A total of 2 × 10^4^ cells were seeded into each insert with serum-free medium, and the low part of the well was filled with 0.6 mL DMEM medium containing 10% FBS. For the peptide treatment, cells were pre-treated with 100 μg/mL C6S-p or scramble peptide for 24 h. The cells were allowed to migrate for 24 h in an incubator. The cells moved to the outer face were stained using crystal violet. Three fields from each filter ere randomly selected to count, and independent experiments were repeated at least three times.

### 2.6. Western Blot

In brief, equivalent amounts of cell lysates (30 μg) were applied to 8 % sodium dodecyl sulfate-polyacrylamide gel electrophoresis (SDS-PAGE), and blotted with antibodies against CD44 (Bioss, BSM51065M), CHSY1 (OriGene, Cat: TA315190, Rockville, MD, USA), CHPF (Proteintech, Cat: 239531AP, Chicago, IL, USA), ITGB1 (BD bioscience, Cat: 610467, Franklin Lakes, NJ, USA) and actin (Santa Cruz, sc-47778). For some experiments, protein lysates were treated with 0.01 U of chondroitinase ABC (ChaseABC, Sigma-Aldrich, Burlington, MA, USA) or 0.1 μg of heparinase II and III (R&D Systems^TM^, Minneapolis, MN, USA) for 2 h at 37 °C to remove the CS or HS chains on the proteoglycan.

### 2.7. Immunoprecipitation, Protein Identification, and Peptide Pulldown Assay

For the immunoprecipitation (IP) assay, cell lysates (0.8 mg) were incubated with 4 µg of antibody at 4 °C for 16 h. Protein L sepharose beads (BioVision Inc., Milpitas, CA, USA) were then added to the lysates for 3 h for pulldown of the CS56 antibody (mouse IgM). For protein identification, pull-downed proteins were digested by trypsin, and applied to tandem mass spectrometry (LC MS/MS, Dionex Ultimate 3000 RSLCnano system hybrid mass spectrometer). The data files obtained following LC-MS/MS analysis were processed in SwissProt, Mascot version 2.5, and Percolator1,2.

Protein A/G sepharose beads (Thermo Fisher Scientific Inc., Waltham, MA, USA) was used to capture CD44 antibody (mouse IgG). The CD44 IP proteins were then separated by 8% SDS-PAGE, and applied to Western blotting. For the C6S-p pulldown assay, a total 0.8 mg of cell lysate was mixed with C6S-p gently and incubated at 4 °C for 16 h. Streptavidin beads (Vector Laboratories, SA-5010, Burlingame, CA, USA) were added to the lysate and incubated for 3 h. The bead pulldown samples were analyzed by Western blotting.

### 2.8. Brain Slice Migration Assay

The brain slice migration assay was conducted according to the previous report [32]. In brief, 8-week-old C57BL/6 wild-type mice were used. The brain tissue was embedded in OCT, and 10μm cross sections of forebrain were pasted on glass slices. A172 cells were labeled with CFSE tracer (Thermo Fisher Scientific Inc., Waltham, MA, USA) for 15 min, and 2000 cells in 1.5 μL culture medium were seeded at the right basal nuclei of each tissue. After cells attached on tissue slide, the culture medium was added to immerse tissue, and the cells were allowed to migrate for 72 h in an incubator. A fluorescence microscope was used to trace and record the cell migration area. For C6S-p treatment, we replaced the medium containing 100 μg/mL C6S-p or scramble peptide, and followed the same process as previously described.

### 2.9. Protein Synthesis Inhibitor Cycloheximide (CHX) Assay

Protein synthesis inhibitor cycloheximide (CHX) was used to stop protein synthesis. The cells were treated with 100 μg/mL C6S-p or scramble peptide, and incubated for 24 h. CHX (20 μM) was treated for 30, 60 or 120 min, and the cell lysates were collected for Western blots.

### 2.10. Reverse-Trancription-PCR

Total RNA was isolated from the cultured cells using a TOOLSmart RNA Extractor (DPT-BD24, Taiwan) following the manufacturer’s instructions. For reverse transcription, first-strand cDNA synthesis was performed with random primers (hexamers; Promega, Madison, WI, USA) and 100 U of moloney murine leukemia virus reverse transcriptase at 42 °C for 60 min and terminated at 90 °C for 10 min. Quantitative real-time PCR analysis was performed using SYBR green one-step PCR Master Mix (Applied Biosystems, Carlsbad, CA, USA) with specific primers (Table S2).

### 2.11. Statistical Analysis

Data analysis was performed using GraphPad Prism 6. CS56 staining intensities and clinic pathologic variables of glioblastoma tissue array were analyzed using the Fisher exact test. The Mann–Whitney *U* test was used to compare staining intensities of unpaired glioma tissues. Bar graphs were used to represent means ± SD, and a *p* value < 0.05 was considered statistically significant.

## 3. Results

### 3.1. Accumulation of CS Is Positively Associated with High Grade Glioma and Predominantly Distributed Surrounding Vasculature

**Figure 1:**
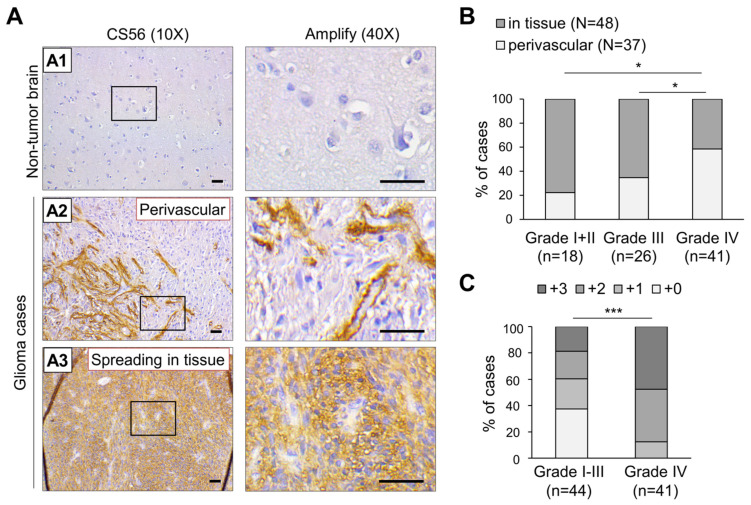
Immunohistochemistry (IHC) analysis of CS56 expression in a tissue array containing primary glioma and non-tumorous brain tissues. (**A**) A total of 85 primary glioma cases and three non-tumor brain tissues on a tissue array were stained for CS56 expression by immunohistochemistry. Staining was visualized as a brown color using the 3, 3-diaminobenzidine (DAB) liquid substrate system. All sections were counterstained with hematoxylin. The CS56 and nuclei staining are depicted by brown and blue colored stains, respectively. Representative images of non-tumor brain (A1), perivascular CS56 staining in glioma tissues (A2), and the spread of CS56 staining in glioma tissue (A3) are shown. Amplified images are shown at right. Scale bars, 100 μm. (**B**) Classification of CS56 distribution patterns in different clinical stages of glioma tissue. Fisher exact test, * *p* < 0.05. (**C**) CS56 staining intensities compared to different stages of glioma tissue. Staining of CS56 was graded by brown area, 0: negative, +1: <20%, +2: 20–50%, +3: >50%. A Mann–Whitney *U* Test was used. *** *p* < 0.001.

To explore the correlation between the distribution of CS and clinical outcomes in glioma patients, an immunochemical analysis with CS56 antibody was performed on a glioma tissue array. The results revealed that non-tumor regions of the normal brain were consistently negative for the expression of CS56 (*n* = 3), while diverse staining patterns were observed in CS56-positive tumor tissue, including intense staining of capillaries, intense staining of adjacent areas surrounding blood vessels, and a weak, moderate, or intense staining that was spread across the tumor tissues (Figure 1A). We further classified the distribution of CS56 into two groups: perivascular (37/85) and spreading in tissue (48/85). Subsequent analyses of the data revealed a significant augmentation of perivascular staining in high-grade gliomas as compared to that in lower grade glioma tissues (Figure 1B). In addition, according to the CS56 staining area of each tissue (negative staining was scored as 0, less than 20% staining was scored as +1, staining between 20–50% was scored as +2, and staining more than 50% was scored +3), the grade IV glioma (glioblastoma; GBM) tissues revealed a higher CS56 staining score than the low-grade glioma tissues (Figure 1C and Table 1). Overall, our results suggest that CS accumulation is elevated in higher grades of glioblastoma and shows frequent distribution in the tissue areas surrounding the vasculature.

### 3.2. CHSY1 Regulates the Expression of Integrins in Glioma Tissues and Cells

**Figure 2:**
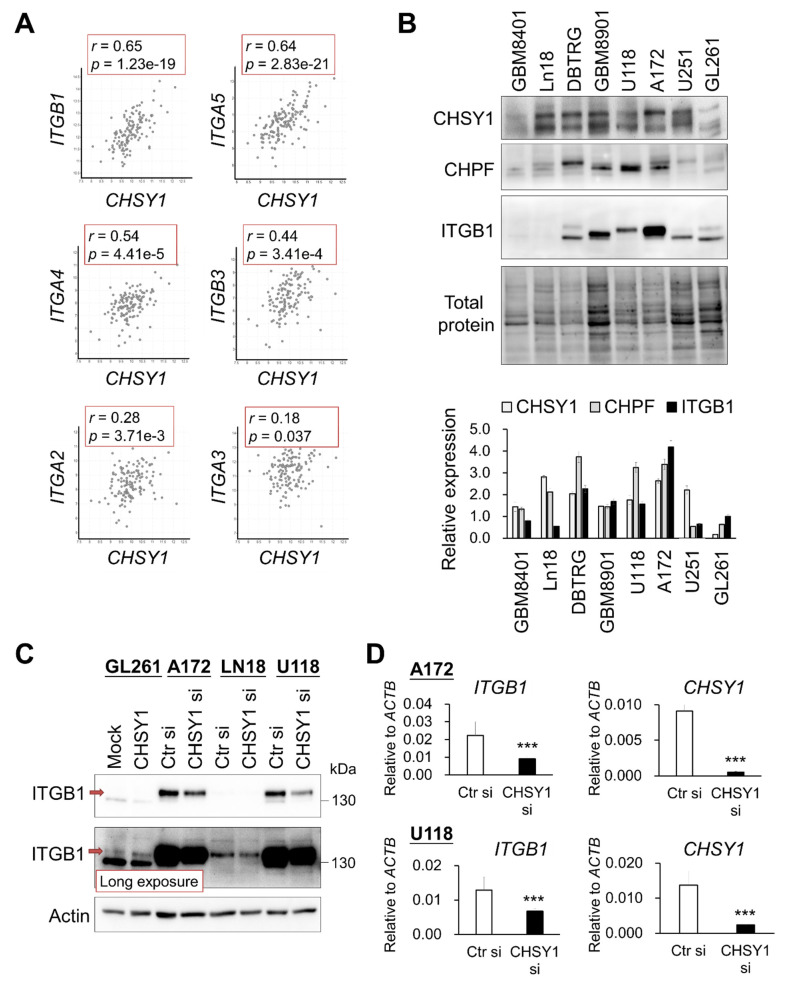
Correlation and modulation between CHSY1 and integrins in glioblastoma tissue and cells. (**A**) Gene expression of CHSY1 in comparison to related integrins. Data were collected from the cBioPortal database and analysed by Pearson’s correlation. (**B**) CHSY1, CHPF, and ITGB1 protein expressions in the glioblastoma cell lines GBM8401, Ln18, DBTRG, GBM8901, U251, A172, U118, and mouse glioblastoma cell line GL261. Each group was standardized with total protein. (**C**) ITGB1 protein expression upon overexpression or silencing of CHSY1 in glioma cells. Control siRNA (Ctr si) or CHSY1-siRNA (CHSY1 si). The red arrow indicates the mature form of ITGB1. (**D**) qPCR analysis of the gene expression of *ITGB1* and *CHSY1* in A172 and U118 cells with *CHSY1* siRNA knockdown. Statistical significance, *** *p* < 0.001.

**Figure 3:**
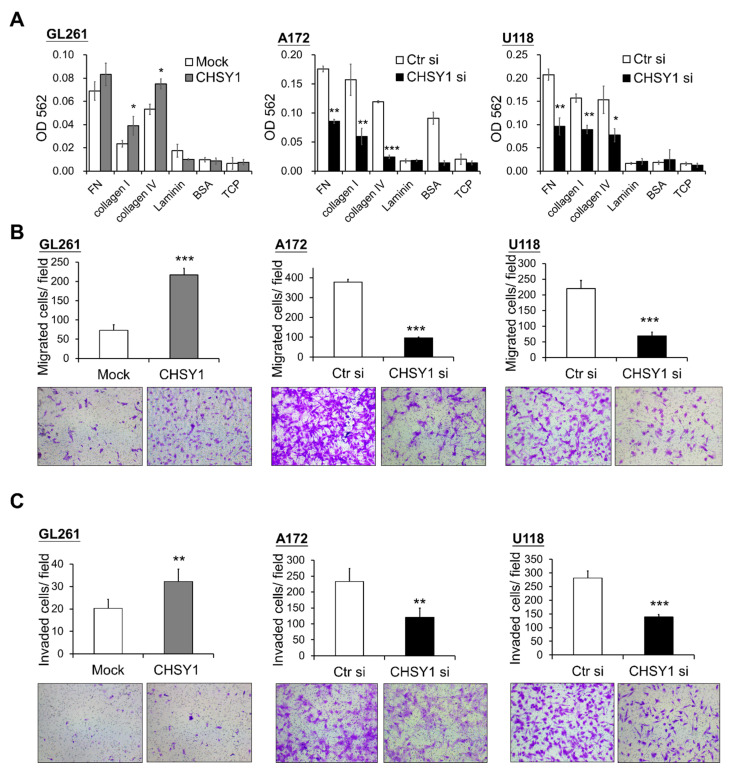
CHSY1 regulates cell adhesion, migration, and invasion in glioma cells. (**A**) Effects of CHSY1 on cell adhesion, (**B**) transwell cell migration, and (**C**) matrigel invasion assays. Stable overexpression of CHSY1 was performed in GL261 cells, and siRNA-mediated knockdown of CHSY1 was performed in A172 and U118 cells. (Ctr si, Control-siRNA; CHSY1 si, CHSY1-siRNA; FN, fibronectin; BSA, bovine serum albumin; TCP, tissue culture plate). Representative micrographs are shown. All results are presented as the means ± SD from three independent experiments. Statistical significance, * *p* < 0.05; ** *p* < 0.01; *** *p* < 0.001.

We have previously demonstrated that CHSY1 functions as the dominant CS synthase in human glioma cells, and its expression is significantly correlated with the anti-CS antibody (CS56) staining intensity in glioma tissues [17]. Thus, to explore the CS-associated pathways in human GBM, we analyzed genes co-expressed with CHSY1 in the TCGA dataset using the cBioPortal for cancer genomics [33]. Intriguingly, the important ECM protein receptors such as integrin β1 (*ITGB1*) and integrin α5 (*ITGA5*), were found to be listed among the top genes co-expressing with *CHSY1* in GBM tissue. Moreover, integrin β3 (*ITGB3*) and integrin α4 (*ITGA4*) also demonstrated a moderately positive correlation with *CHSY1* (Figure 2A). Our analyses further revealed that the expression of other CS synthases, such as *CHPF*, showed weak or no correlation with these integrins (Figure S1 and data not shown). To examine the expression of CS synthases and ITGB1 at the protein level, the human glioma cell lines GBM8401, Ln18, DBTRG, GBM8901, U118, A172, U251, and a mouse GBM cell line GL261 were analyzed by Western blotting. The results showed that both ITGB1 and their CS synthases CHSY1 and CHPF were highly expressed in DBTRG, GBM8901, U118, and A172 cells as compared to other cells examined in the study (Figure 2B). To verify the correlation between CHSY1 and integrin β1, we silenced the expression of CHSY1 by using specific-siRNA in Ln18, A172, and U118 cells, which expressed relatively high to moderate levels of CHSY1; and overexpressed CHSY1 in GL261 cells, which expressed relatively low levels of CHSY1. The Western blotting studies revealed that silencing CHSY1 evidently decreased the expression of ITGB1, whereas its expression was increased in cells overexpressing CHSY1 (Figure 3C). Additionally, we found that knockdown of CHSY1 decreased the mRNA expression of *ITGB1*, indicating that it is involved in regulating the expression of *ITGB1* at the transcriptional level (Figure 2D).

### 3.3. CHSY1 Regulates Invasive Potential of Glioma Cells

As integrins are crucial mediators of cell and ECM protein interactions, we hypothesized that they may participate in regulating the CS-induced malignant phenotypes in glioma cells. Accordingly, cell adhesion assays were used to assess the glioma cell attachment to ECM proteins in vitro. The results demonstrated that overexpression of CHSY1 enhances GL261 cell adhesion to collagen I and IV, while silencing CHSY1 led to a decrease in cell attachment to fibronectin, collagen I, and collagen IV as compared to respective control cells (Figure 3A). Furthermore, transwell migration assays were performed to determine glioma cell mobility and invasiveness. Consistent with the cell adhesion assay results, the overexpression of CHSY1 significantly increased the cell invasion and migration of GL261 cells, whereas the knockdown of CHSY1 evidently suppressed the invasion and migration of A172 and U118 cells (Figure 3B,C).

### 3.4. CD44 Is a CHSY1-Modified CSPG in Glioma Cells

**Figure 4:**
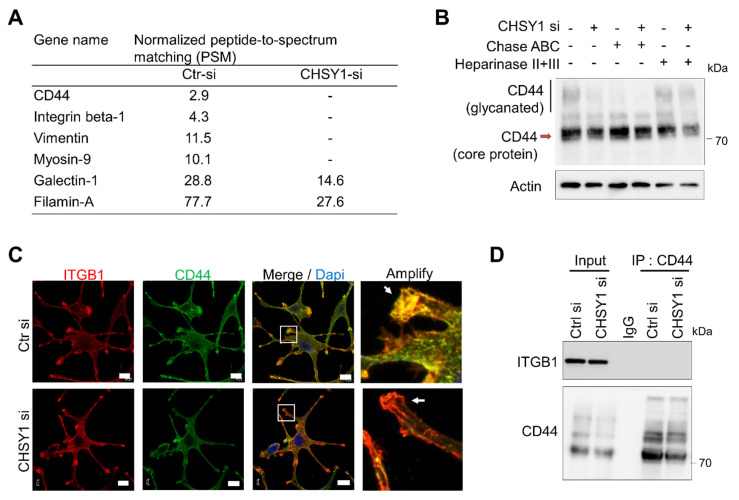
CHSY1 modulates the CS chains on CD44. (**A**) List of peptide-to-spectrum matching (PSM) analysis of control A172 cell lysate (Ctr si) and CHSY1 silenced cell lysate (CHSY1 si) after immunoprecipitation by CS56 antibody. CSPG and focal adhesion-related protein are shown. (**B**) Western blots of CD44 on control (Ctr si) and CHSY1 silenced cell lysates (CHSY1 si) after treatment with chondroitinase ABC (Chase ABC) or heparinase. (**C**) Confocal microscopy analysis of ITGB1 (red) and CD44 (green) distribution around the cell surface. Scale bar, 8 μm. Amplified images are shown at right. (**D**) Immunoprecipitation (IP) of CD44 and immunoblots with ITGB1 and CD44 antibodies.

Our study so far revealed that the critical CS synthase, CHSY1, regulates the expression of integrins and cell invasive phenotypes in glioma cells. Hence, we aimed at identifying the particular CSPGs involved in this regulation. Accordingly, immunoprecipitation assays with CS56 antibody and subsequent mass spectrometry-based protein identification were performed in the control and CHSY1-silenced A172 cell lysates. The results showed that only CD44 was detected in the CS56 antibody-mediated CSPG pulldown of the control cell lysates. Interestingly, several focal adhesion-associated proteins including integrin β1, vimentin, and Myosin-9 were also detected in the control cell lysates (Figure 4A). As CD44 can bear heparan sulfate (HS) or CS accumulation depending on the cellular enzyme expression profile, chondroitinase ABC or heparinase were used to remove the CS or HS chains on the glycanated CD44, respectively. Subsequent Western blotting studies revealed that glycanated CD44 is significantly decreased post treatment with chondroitinase ABC. However, heparinase treatment did not show evident effects on the levels of glycanated CD44 in A172 cells (Figure 4B). Furthermore, the knockdown of CHSY1 also decreased the expression of glycanated CD44 and slightly decreased the total CD44 expression. Several studies have indicated that CD44 cooperates with integrins to regulate cancer cell adhesion and mobility [34,35,36]. Consequently, we analyzed the locations of integrin β1 and CD44 using confocal microscopy. Our results revealed that the integrins β1 and CD44 were primarily assembled on the pseudopodia in the control cells, whereas the same phenomenon was found to be disrupted upon the silencing of CHSY1 (Figure 4C). However, our CD44 co-immunoprecipitation assays failed to detect direct protein binding between ITGB1 and CD44 under our experimental conditions (Figure 4D). Together, these results suggest that CD44 functions as a CSPG in glioma cells, and that CS chains may be involved in facilitating CD44 and integrin cooperation on adhesion complexes.

### 3.5. C6S Binding Peptide Interrupts Invasive Phenotype of Glioma Cells

**Figure 5:**
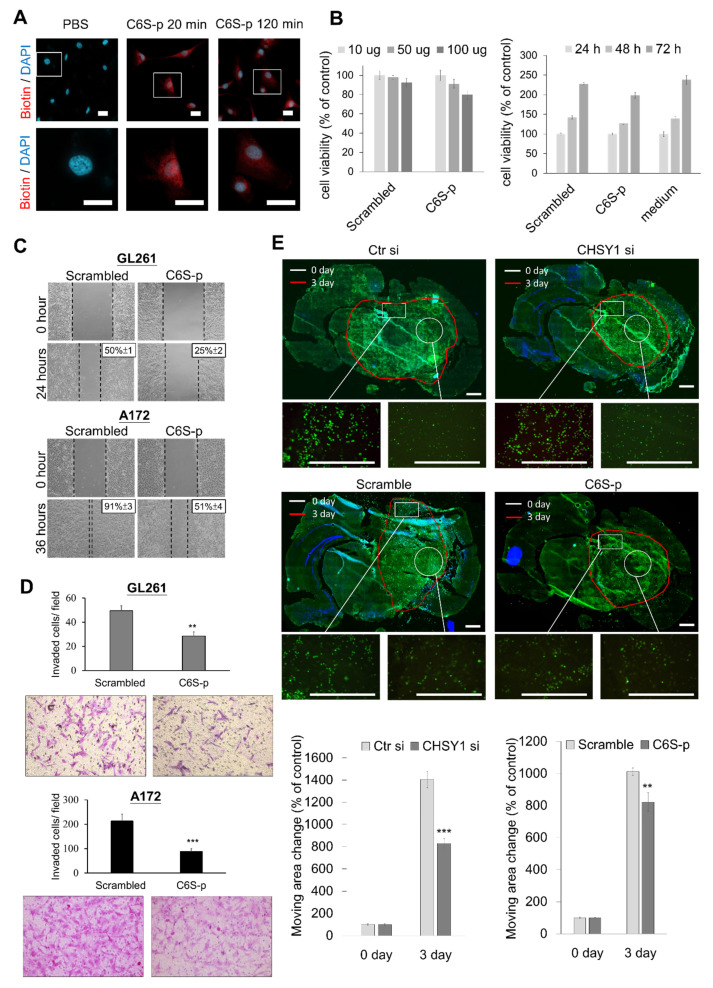
Effects of CS-binding peptide (C6S-p) on glioma cells. (**A**) Immunocytochemistry of biotin staining with streptavidin-Alexa 594 (red) and nuclear with DAPI (blue). A172 cells were treated with PBS or C6S-p for 20 min or C6S-p for 120 min. Scale bar, 12.5 μm. Amplified images are shown at lower panel. (**B**) A CCK8 assay was used for detecting the effect of C6S-p on the viability of A172 cells. Treatment with different doses (10, 50, and 100 μg) of C6S-p or the control scrambled peptide for 24 h (left); and the time course treatment with 100 μg of C6S-p or control scrambled peptide for 24, 48, and 72 h (right). Cell viability is represented as percentage relative to the control group. (**C**) Scratch assays in GL261 and A172 glioma cells treated with C6S-p or the scrambled peptide; the average area of wound closure is shown as a percentage relative to control. (**D**) Matrigel invasion assays in GL261 and A172 glioma treated with C6S-p or the scrambled peptide; migrated cells are shown as the number of invaded cells per field. (**E**) Examining the ex vivo migration of A172 cells transfected with control siRNA (Ctr si) or CHSY1-siRNA (CHSY1 si, upper) and A172 cells treated with scrambled peptide or C6S-p (lower) on mouse brain tissue slice. The white circles indicate the original location of the cell seeding at day 0, and the red line indicates the cell moving edge after 72 h. Representative images are shown. Scale bars, 150 μm. The mean ± SD of area changes from four independent experiments were shown at bottom. Statistical significance, ** *p* < 0.01; *** *p* < 0.001.

The CS56 antibody preferentially binds to 6-O-sulfated CS (C6S) [37,38]. To evaluate the effects of targeting C6S on glioma, we reviewed the C6S binding peptides which have been proven to block the activity of C6S [29,30,31]. We tested a C6S-specific binding peptide (C6S-p, biotinylated), which revealed high affinity to C6S and rescued the inhibition of C6S on neurite outgrowth. Our in vitro studies showed that C6S-p binds to the glioma cell surface under culture conditions (Figure 5A). Subsequent assessment of cell viability to evaluate the cytotoxicity of C6S-p using the CCK8 assay revealed that C6S-p treatment slightly decreased, but did not significant affect, the viability of A172 cells in both dose- and time-dependent experiments (Figure 5B). To determine the effect of C6S-p on the mobility of glioma cells, scratch healing assays were performed. The results of these assays showed that the C6S binding peptide inhibited the cell migration for recovering the scratch in both GL261 and A172 cells as compared to the scramble peptide control; the recovered area in the scramble peptide control treatment vs. C6S binding peptide treatment was 50% versus 25% for the GL261 cells and 91% versus 51% for the A172 cells, respectively (Figure 5C). Consistent inhibitory effects of C6S-p were also observed in the transwell invasion assays (Figure 5D). Further assessment of the effects of CS on ex vivo CNS tissues was performed using the brain slice migration assays. The results revealed that both the CHSY1 knockdown and C6S-p treatment groups significantly suppressed the migration area of CNS cells in the brain slice migration assays (Figure 5E).

### 3.6. C6S Binding Peptide Triggers CD44 Degradation and Suppresses ITGB Expression

**Figure 6:**
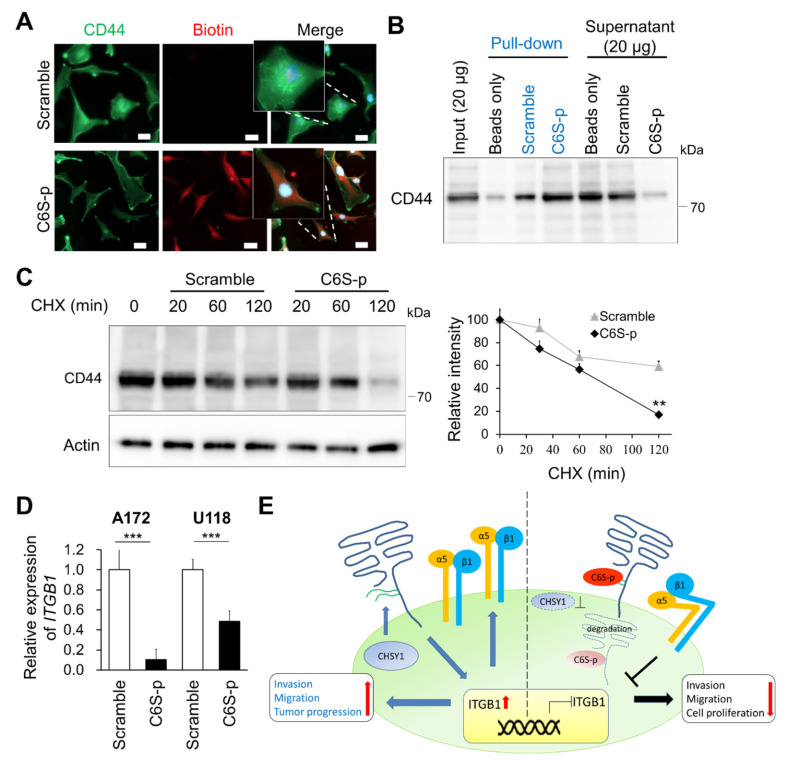
C6S-p binds to CD44 and accelerates CD44 degradation. (**A**) Co-localization between CD44 and C6S-binding peptides. Peptides staining with biotin (red) and CD44 antibody (green). Scale bar 8 μm. Amplified images are shown. (**B**) Streptavidin beads pulldown assay isolated the CD44-C6S-p complex. A172 cell lysate was used for the biotinylated peptide pulldown assay. Protein levels of CD44 were analyzed by Western blotting. (**C**) Western blotting of CD44 degradation. A172 cells were treated with peptides and 20 μM cycloheximide (CHX) for 30, 60 and 120 min. Actin was used as the loading control. The relative protein levels are shown on the right. ** *p* < 0.01. (**D**) Real-time PCR analysis of *ITGB1* mRNA expression after treatment with C6S-p and scramble peptide in A172 and U118 cells. *** *p* < 0.001. (**E**) A proposed model illustrating the co-operation of CD44 with integrins to enhance glioma cell-invasive phenotypes. The model also demonstrates that the knockdown of CHSY1 or blocking CS on CD44 may promote the degradation of CD44 and suppress the expression of integrins in glioma cells.

Our findings have shown that C6S-p can target 6-O-sulfated CS and has similar effects as that of CHSY1 knockdown. We further studied whether C6S-p influences the levels of CD44. Accordingly, our immunocytochemistry studies revealed that C6S-p and CD44 co-localize on the cell surface (Figure 6A). Moreover, the staining intensity of CD44 was determined to be slightly decreased in C6S-p treated A172 cells. Subsequent co-immunoprecipitation assays employing streptavidin beads to pull down biotin-C6S-p confirmed that C6S-p evidently binds to CD44, as a large proportion of CD44 in the A172 cell lysate was pulled down by C6S-p (Figure 6B). To examine whether C6S-p promotes CD44 degradation, the protein synthesis inhibitor cycloheximide (CHX) was used to stop protein translation, and the CD44 protein levels were determined subsequently (Figure 6C). The results revealed that CD44 expression was significantly lower in the C6S-p targeted A172 cells post 2 h of CHX treatment as compared to the untreated control cells (*p* < 0.01). Furthermore, C6S-p treatment also resulted in the suppression of *ITGB1* mRNA expression in glioma cells as compared to non-treated glioma cells (Figure 6D), which is consistent with the effects of CHSY1 silencing using specific siRNAs (Figure 2D). Overall, these findings suggest that C6S-p regulates the expression of ITGB1 by blocking the CS chain on CD44.

## 4. Discussion

Accumulating evidence has revealed that the aberrant expression of CS synthases and CS modification enzymes along with increased CSPGs in the tumor microenvironment are characteristic hallmarks of several types of cancer, including glioma [17,39,40,41,42,43,44]. We have demonstrated that the staining intensity of CS56 is positively associated with high-grade glioma using immunohistochemistry on a glioma tissue array, which is consistent with the results of a recent study that has demonstrated that CS accumulation is augmented in another independent glioma cohort [45]. Interestingly, we have demonstrated CS56 staining of the perivascular area for the first time in a subset of glioma tissue, in addition to the depiction of a certain vascular structure. In contrast, this staining pattern was undetectable in the vessels of normal brain tissue. The vasculature in glioma has several functions that enable tumor growth, such as the delivery of oxygen and nutrients, modulation of the immune response, and facilitation of tumor cell dissemination. Notably, tumor-initiating cells (TICs) or cancer stem cells (CSCs) are considered to be localized at the perivascular niche in glioma tissues [46,47], while CD44 and CSPG4 serve as critical markers of TIC [48]. Additionally, glioblastoma CSCs have been reported to transform into vascular pericytes [49], and angiogenic vessels in GBM tissue demonstrate the expression of CSPG4 [13,50]. Thus, our data suggest that the perivascular staining of CS56 could be an indicator for TIC-rich areas in glioma. Accordingly, it is worth investigating whether C6S-p is capable of targeting the TIC in glioma tissue.

The transmembrane proteoglycan, CD44, is a hyaluronic acid (HA) receptor which is expressed in several healthy and tumor tissues. It is not only involved in cell–cell interactions and cell adhesion, but it is also considered a CSC marker in certain types of cancer [51]. However, the functions of the GAG chains on CD44 are still not well understood. It has been reported that removal of cell surface GAG by treating cultured cells with β-D-xylopyranoside or chondroitinase ABC inhibits the HA-binding function of CD44 [52]. In our study, we found that silencing CHSY1 in cultured GBM cells results in the attenuation of both glycanated CD44 and core total CD44 protein, as well as decreased CD44 expression on the pseudopodia of cells (Figure 4B). Moreover, treating glioma cells with a C6S binding peptide also enhances the turnover of CD44. Although these approaches affect all CSPGs on cancer cells, our data suggest that CS chains can directly or indirectly modulate CD44 stability on the surface of glioma cells.

The interaction between CD44 and integrins which mediate cancer cell mobility has been documented in multiple studies [53,54,55]. In particular, integrin β1 is tightly coupled to CD44 in lipid rafts and triggers signaling to govern cellular movement and survival [36,55]. In this context, the failure to detect integrin β1 in the CD44 immunoprecipitation assay may be attributed to the fact that the detergent-resistant lipid raft proteins were not enriched from cultured glioma cells in the present study (Figure 4D). Nevertheless, our confocal microscopy images confirmed that CD44 and integrin β1 are largely co-localized on the invasive fronts of control glioma cells. In contrast, knockdown of CHSY1 evidently decreased the co-localization of integrin β1 and CD44, which may explain the associated attenuation of cellular signaling from the cell adhesion complex.

Our TCGA data analyses suggest the presence of a positive correlation between integrins and CHSY1 in GBM patients. Further in vitro manipulation of CHSY1 expression has been shown to alter the expression of ITGB1 at both the translational and transcriptional levels. These findings suggest that CHSY1-mediated signaling regulates the expression of integrins in glioma cells. It has been reported that CD44 signaling rapidly stimulates integrin α5 and β1 expression and activation in breast and prostate cancer cells [56]. We thus hypothesized that targeting CS chains by knockdown of CHSY1 or treating glioma cells with C6S binding peptides may result in decreased CD44 protein levels, and inhibit the outside-in signals from regulating the gene expression of integrins. Additionally, the interaction between CD44 and integrins, documented to coordinate the downstream Akt, Erk, and Src signaling, may further induce the expression and activation of integrins [55,57]. Thus, the overall findings of our study conclude that CHSY1-mediated CS may promote CD44 activity and consequently regulate integrin expression in glioma cells, thereby serving as a potential pathway for therapeutic targeting in glioma.

## 5. Conclusions

GBM is one of the most aggressive human cancers, with very high mortality. The high mortality rate of GBM is mainly attributable to the diffuse infiltration of tumor cells into the brain tissue, causing its inevitable recurrence after surgical resection. Thus, strategies for suppressing glioma cell invasiveness hold remarkable potential for the development of novel therapies for the treatment of GBM. Our study has demonstrated that CHSY1 regulates CS accumulation in association with CD44 on the cell membrane and modulates ITGB1 mRNA expression. Moreover, our attempts to employ a C6S specific binding peptide to simulate the blocking of CS functions led to similar results as those observed for CHSY1 knockdown (Figure 6E). Based on these studies, it is worth further investigating the tumor suppressive effects of C6S-p in vivo. These studies may thus offer beneficial insights, highlighting a novel strategy for the development of effective GBM therapeutic agents in the future.

## Figures and Tables

**Table 1:** Correlation of CS56 staining with clinicopathological features of glioma tissue array.

		CS56 Intensity		*p* Value (Two-Sided Fisher Exact Test)
Factor		Low (0 and +1)	High (+2 and +3)	
Tissue types	Non-tumor	3	0	0.05
	Tumor	31	54	
Gender ^#^	Male	10	32	0.017
	Female	18	17	
Age ^#^	<55 years	15	19	0.239
	≥55 years	13	30	
Tumor stage	Grade I–III ^$^	27	18	<0.0001
	Grade IV (GBM)	4	36	

^#^ 8 patients’ gender and age were not provided. ^$^ Astrocytoma and oligodendroglioma.

## Data Availability

The data that support the findings of this study are openly available.

## Supplementary Materials

The following are available online at https://www.mdpi.com/article/10.3390/cells10123594/s1, Table S1. Information of glioma cell lines. Table S2. Quantitative PCR primer and siRNA sequence list. Figure S1. Correlation of CHPF and integrins expression in human glioblastoma cases. Figure S2. Original images of western blots.
