# Supplementary material for: Targeting Chondroitin Sulfate Reduces Invasiveness of Glioma Cells by Suppressing CD44 and Integrin β1 Expression
Source: Cells. 2021 Dec 20;10(12):3594. doi: 10.3390/cells10123594 (PMC8700349; doi:10.3390/cells10123594)
Supplement: Supplementary file 1 [file cells-10-03594-s001.zip › cells-1485324-supplementary.pdf]

Table S1. Information of glioma cell lines.

Name	Database Name/ Accession Numbers	Domain	Source
Ln18	ATCC / CRL-2610	Cell Line	Homo sapiens (human) brain; glioblastoma
DBTRG-05MG (DBTRG)	Bioresource Collection and Research Center (BCRC), Taiwan / BCRC Number: 60380	Cell Line	Homo sapiens (human) brain; glioblastoma
U251-MG (U251)	Kerafast, Inc. / Number: EDK001	Cell Line	Homo sapiens (human) brain; glioblastoma
A172	ATCC / CRL-1620	Cell Line	Homo sapiens (human) brain; glioblastoma
U-118 MG (U118)	ATCC / HTB-15	Cell Line	Homo sapiens (human) brain; glioblastoma
GBM8401	Obtained from Dr. Wei-Hwa Lee, Tri-service General Hospital, Taipei, Taiwan. BCRC Number: 60163	Cell Line	Homo sapiens (human) brain; glioblastoma
GBM8901	Obtained from Dr. Wei-Hwa Lee, Tri-service General Hospital, Taipei, Taiwan. BCRC Number: 60164	Cell Line	Homo sapiens (human) brain; glioblastoma
GL261	Leibniz Institute DSMZ / Number: ACC 802	Cell Line	Mus musculus (Mouse) Breed/subspecies: C57BL/6. Mouse glioblastoma

Table S2. Quantitative PCR primer and siRNA sequence list

Symbol	Gene name	Used	Sequence (5'-3')
CHSY1	Chondroitin Sulfate Synthase 1	qPCR	F: GGCACGCGTATTTACAGCAG
		qPCR	R: TTGTGCTCACTCTTCGACCC
ITGB1	Integrin Subunit Beta 1	qPCR	F:GCCGCGCGGAAAAGATG
		qPCR	R:GAATTTGTGCACCACCCACAA
ACTB	Actin, beta	qPCR	F: CACCATTGGCAATGAGCGGTTC
		qPCR	R: AGGTCTTTGCGGATGTCCACGT
CHSY1	Chondroitin Sulfate Synthase 1	siRNA	GGCAAGUGUCUCCGGGAAA
		siRNA	AUUCCAAAUCUGAACGUGA
		siRNA	GACAAGUACGAAUGGUUUA
		siRNA	GAGAUUACCGAGUCAAGUA
	Non-target	siRNA	UGGUUUACAUGUCGACUAA
		siRNA	UGGUUUACAUGUUGUGUGA
		siRNA	UGGUUUACAUGUUUUCUGA
		siRNA	UGGUUUACAUGUUUUCCUA

Figure S1.

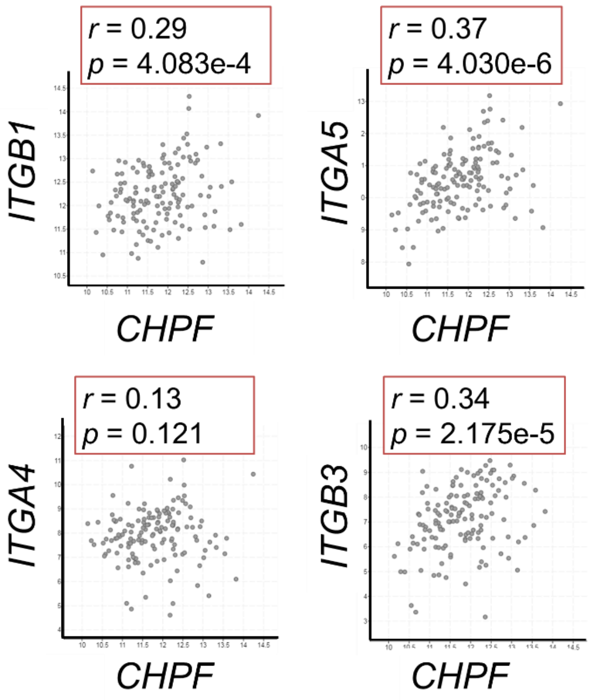

Figure S1. Correlation of *CHPF* and integrins expression in human glioblastoma cases. *CHPF* gene expressions compared with *ITGB1*, *ITGB3*, *ITGA4*, and *ITGA5*. Data were collected from cBioPortal database, and analyzed by Pearson's correlation.

Figure S2.

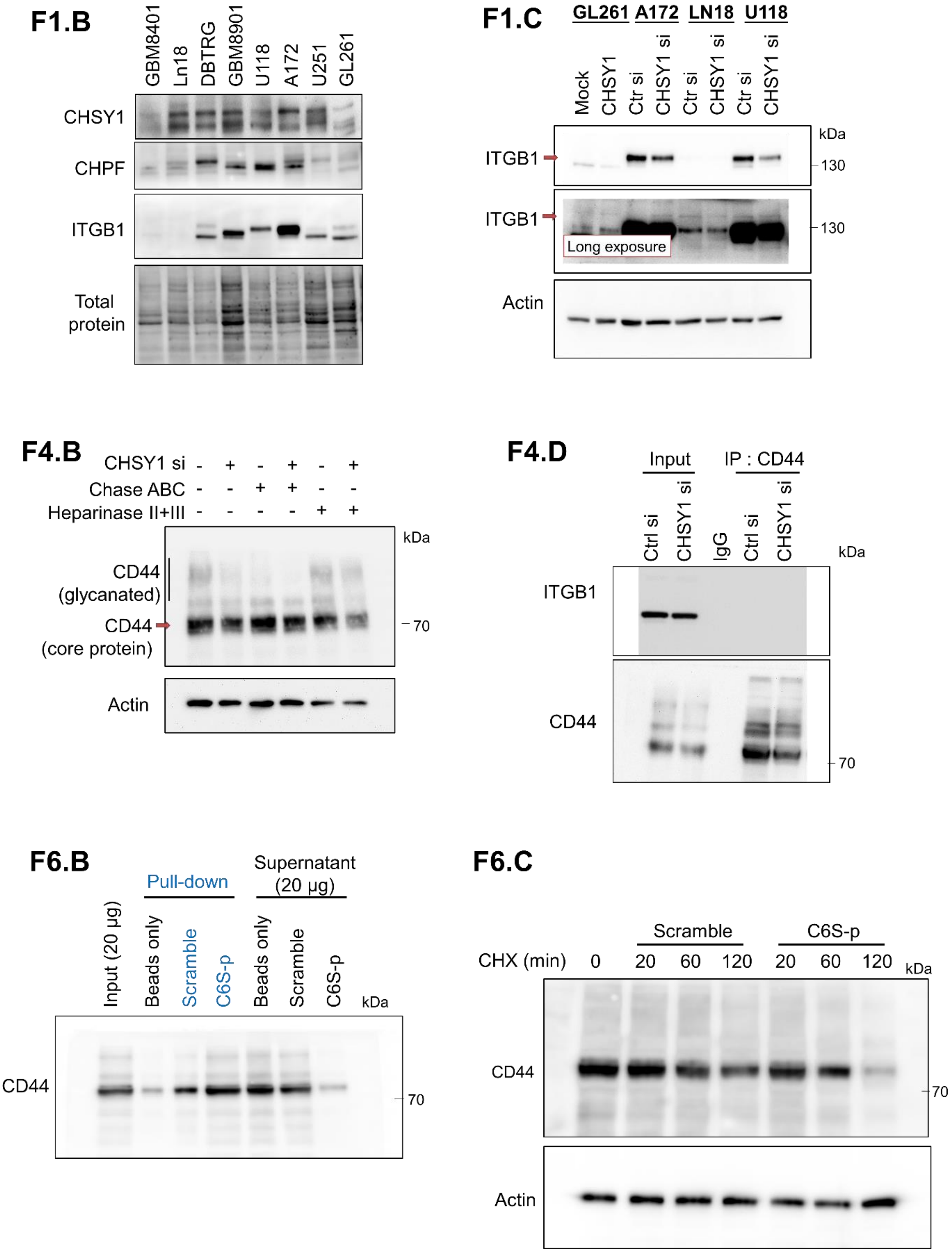

Figure S2. Original images of western blots
